# Comparing the Effects of Nocturnal Sleep and Daytime Napping on Declarative Memory Consolidation

**DOI:** 10.1371/journal.pone.0108100

**Published:** 2014-09-17

**Authors:** June C. Lo, Derk-Jan Dijk, John A. Groeger

**Affiliations:** 1 Cognitive Neuroscience Laboratory, Duke-NUS Graduate Medical School, Singapore, Singapore; 2 Surrey Sleep Research Centre, University of Surrey, Guildford, United Kingdom; 3 Department of Psychology, University of Surrey, Guildford, United Kingdom; 4 Department of Psychology, University of Hull, Hull, United Kingdom; Catholic University of Sacred Heart of Rome, Italy

## Abstract

Nocturnal sleep and daytime napping facilitate memory consolidation for semantically related and unrelated word pairs. We contrasted forgetting of both kinds of materials across a 12-hour interval involving either nocturnal sleep or daytime wakefulness (experiment 1) and a 2-hour interval involving either daytime napping or wakefulness (experiment 2). Beneficial effects of post-learning nocturnal sleep and daytime napping were greater for unrelated word pairs (Cohen’s *d* = 0.71 and 0.68) than for related ones (Cohen’s *d* = 0.58 and 0.15). While the size of nocturnal sleep and daytime napping effects was similar for unrelated word pairs, for related pairs, the effect of nocturnal sleep was more prominent. Together, these findings suggest that sleep preferentially facilitates offline memory processing of materials that are more susceptible to forgetting.

## Introduction

Sleep facilitates consolidation of declarative memory [1] such that recall of previously acquired materials is better after sleep than after wakefulness. These effects are observed for post-learning sleep at night [2], [3] and naps during the daytime [4], [5], but a direct comparison of these effects is currently not available. Besides the difference in circadian phase, nocturnal sleep and daytime naps differ in multiple important aspects, such as total sleep time, the amount of sleep spindle-rich stage 2 sleep, slow wave sleep (SWS), and rapid eye movement (REM) sleep. These differences might contribute to differential facilitative effects of nocturnal sleep and daytime napping on declarative memory consolidation [6].

Furthermore, nocturnal sleep and daytime naps may not facilitate consolidation of all declarative materials equally. In studies that used either semantically related [4], [7] or unrelated [5], [8] word pairs, superior recall of both was found after both nocturnal sleep and daytime naps relative to wakefulness. However, in a study which directly tested the moderating effects of semantic relatedness, nocturnal sleep only abolished the forgetting of unrelated word pairs observed over wakefulness [3]. These differential effects have not been investigated for daytime naps.

Here, in experiment 1, we attempted to replicate the differential effects of nocturnal sleep on semantically related and unrelated word pairs. In experiment 2, we quantified the effects of a 90-minute post-learning daytime nap on these two kinds of materials. Using effect size measures, we then compared whether nocturnal sleep and daytime napping benefited related and unrelated materials to different extents.

## Method

### Participants

Sixty young adults aged 18–35 years participated in experiment 1 (mean age ± SD = 21.9±4.2 years; 17 males) and 34 in experiment 2 (21.9±2.8 years; 11 males). Participants reported a habitual bedtime of 22∶30–01∶00, wake time of 06∶30–08∶30, and sleep duration of 5–9 hours. They did not report extreme morningness/eveningness preference, persistent sleep difficulties, or taking any medication, except oral contraceptives. Participants in experiment 2 napped at least once every week.

### Procedures

In both experiments, compliance to habitual sleep-wake schedule the night before each experimental session was verified with actigraphy (Actiwatch-L; Cambridge Neurotechnology). Participants abstained from caffeine, alcohol, and napping 24 hours before each session.

#### Experiment 1

In experiment 1, we used a between-subject design. Participants were randomly assigned to the sleep group (n = 30) and the wake group (n = 30). For the sleep group, learning started at 21∶00 and retest at 09∶00 the following day. For the wake group, learning and retest were at 09∶00 and 21∶00 respectively on the same day. Napping was not allowed during the retention interval, and was verified with actigraphy.

In the paired-associate task, 80 cue-target word pairs, taken from an earlier study [9], were presented sequentially on a computer screen for 3,000 ms each, with an inter-stimulus interval of 500 ms. Cues were always displayed on the left side of the screen and targets on the right. Each of the three presentation blocks was immediately followed by a cued recall test in which participants were shown only the cue of each pair for 2,000 ms, and they needed to say aloud the target word with which each was originally paired. A cued recall test was also administered at the retest session.

Among the 80 word pairs, 40 were semantically related to each other (e.g. pilot-plane), while the others were semantically unrelated (e.g. pepper-elbow). The difference in semantic relatedness between these two kinds of word pairs was validated in a pilot study (N = 10) in which participants rated each pair on how well the two words were related to each other in meaning (“0”  = “very unrelated”; “4”  = “very related”). They attributed significantly higher ratings to the *a priori* defined related pairs (3.44±0.21 vs. 0.92±0.54, *t* = 26.37, *P*<0.001). In fact, these pairs had the highest 40 semantic relatedness ratings, while the lowest 40 ratings were given to the *a priori* defined unrelated word pairs.

#### Experiment 2

In experiment 2, we used a within-subject design. Learning began at 13∶30 on average. In the nap condition, learning was followed by a 90-minute napping opportunity with polysomnographic monitoring. Upon awakening, retest started after a 30-minute computerized puzzle game to minimize influence of sleep inertia. In the wake condition, participants played this game throughout this 2-hour period. Participants attended 4 laboratory sessions at least 5 days apart. We manipulated physiological state (nap vs. wake) and learning strategies (strategy to boost learning vs. control in one sub-experiment, and strategy to reduce learning vs. control in another sub-experiment). The order of conditions was counterbalanced across participants. Here, to increase the comparability with experiment 1, we only report findings from the control conditions where learning strategies were not imposed.

In the paired-associate task, similar to experiment 1, participants learned 40 semantically related and 40 semantically unrelated word pairs in each laboratory session, but a new list was used each time. The word pairs used in experiment 2 were different from experiment 1, since there were four experimental sessions and more learning materials were required. Word pairs were extracted from the University of South Florida Free Association Norms database [10]. Word pairs were initially selected if their target word had been normed, both their cue and target words were concrete (concreteness rating 3.5 on a 7-point scale) and were common words (frequency of occurrence 20 times per million). A total of 518 word pairs fulfilled these criteria. One hundred and sixty pairs that had the highest pre-existing associations, as indicated by their forward cue-to-target strength (the percentage of individuals giving the target word upon being presented with the cue), and did not have any overlapping cue or target words were selected as the semantically related word pairs. Their forward cue-to-target strength ranged from 0.28 to 0.89. To generate the semantically unrelated word pairs, the remaining 358 word pairs were split up and any word that was already the cue or the target of the related word pairs was removed. The remaining 356 words were randomly paired up and the first 160 pairs were chosen as the semantically unrelated word pairs.

In a pilot study, 10 individuals provided a semantic relatedness rating for each pair on a 5-point Likert scale (“0”  = “very unrelated”; “4”  = “very related”). The ratings on the *a priori* defined related pairs were significantly higher than that on the *a priori* defined unrelated pairs (3.69±0.28 vs. 0.50±0.52, *t* = 26.37, *P*<0.001). The four lists did not differ in semantic relatedness (related word pairs: *F* = 0.32, *P*>0.05; unrelated word pairs: *F* = 1.83, *P*>0.05).

In each learning block, the cue of the word pair was first presented on a computer screen for 1,000 ms each, followed by the cue and the target simultaneously for 3,000 ms. Afterwards, the target was presented for 1,000 ms during which the participants were required to say aloud the target word. The inter-pair interval was 1,000 ms.

Each learning session consisted of three learning blocks, each of which was followed by an immediate cued recall test where only the cue of each pair was shown for 1,000 ms. Participants were given the next 2,000 ms to provide a verbal response. After a 500-ms interval, the cue of the next pair was presented and so on. In the retest session, paired-associate memory was assessed with a cued recall test. In all the learning blocks and during all the recall tests, word pairs were presented randomly.

In both experiments, participants were instructed to learn as many word pairs as possible. They were informed that their memory would be tested after the retention interval. The cued recall tests used in both learning and retest did not involve any feedback. Participants’ verbal responses were recorded and subsequently coded manually. Learning performance was indicated by the number of word pairs correctly recalled at the end of learning. Change in the number of correct recalls from the end of learning to retest was used as a measure of memory consolidation.

We conducted power analysis to determine the sample size required for the two experiments. In both cases, we set alpha at 0.05 (two-tailed) and power at 0.90. For experiment 1, to detect a significant effect of nocturnal sleep on memory consolidation, 26 participants would be required for each of the two groups based on a Cohen’s *d* of 0.92 [11]. For experiment 2, to detect a significant nap effect with a within-subject design, 18 participants would be required based on a Cohen’s *d* of 0.79 [4] for each of the two sub-experiments where we manipulated different learning strategies.

### Polysomnography

Sleep EEG during the 90-minute nap opportunities in experiment 2 was recorded with a Vitaport 3 recorder (TEMEC) and a ten-channel montage consisting of six EEG (F3-A2, F4-A1, C3-A2, C4-A1, O2-A1, O1-A2), two EOG, and two EMG submental channels. The sampling rate and the storage rate were 256 Hz. The low-pass filter was set at 70 Hz and the high-pass filter was set at 0.3 Hz. Electrode impedance was kept below 5kΩ. Each 30-second epoch was scored according to Rechtschaffen and Kales criteria [12].

These experiments were approved by the University of Surrey Ethics Committee and were conducted at the Department of Psychology of the University of Surrey in accordance with the principles of the Declaration of Helsinki. All the participants provided written informed consent after receiving a detailed explanation of the aims and procedures of the study. All the data used in this study were anonymized.

### Statistical Analyses

Statistical analyses were performed with SAS 9.1 (SAS Institute, Cary, NC). We used a general linear mixed model with PROC MIXED which included Group (experiment 1) or Condition (experiment 2) as a fixed effect to determine whether learning performance differed between the sleep and the wake groups/conditions. Relatedness was included as a repeated effect to determine whether acquisition of related and unrelated word pairs differed, and a compound symmetry matrix was specified. Their interaction was also included.

For experiment 2, in addition to the above, Period was added as a fixed effect to examine whether performance was affected by prior exposure to the task and thus, varied across the four experimental sessions. Also, subject effect was included as a random factor.

The same statistical model was used on retest performance and memory consolidation. Differences of least square means were used to determine significant differences between the two groups/conditions. Effects of nocturnal sleep and daytime napping were quantified with Cohen’s *d* with 0.2, 0.5, and 0.8 as cut-offs for small, medium, and large effect sizes respectively [13]. Performance changes across the retention interval were tested against 0 to determine whether forgetting was significant. Independent-samples *t* tests were used to compare least square means of performance changes across nocturnal sleep and across a daytime nap, as well as across 12 and 2 hours of wakefulness.

In experiment 2, we used Pearson correlations to determine whether the duration and the macrostructure, i.e. the duration of the various sleep stages, of the post-learning nap episode were significantly associated with the performance change across the retention interval.

## Results

### Experiment 1: nocturnal sleep

Participants were better at learning related than unrelated word pairs (Relatedness: *F_1,58_* = 191.30, *P*<0.001). Although learning was at different times of day, the sleep and the wake groups showed similar learning performance (Group: *F_1,58_* = 0.38, *P* = 0.54; Relatedness×Group interaction: *F_1,58_* = 0.43, *P* = 0.51; Table 1).

**Table 1:** Effects of nocturnal sleep and daytime napping on paired-associate performance.

	Experiment 1 (12-hour retention interval)					Experiment 2 (2-hour retention interval)				
	Sleep	Wake	*t_58_*	*P*	*d*	Nap	Wake	*t_33_*	*P*	*d*
*Learning*										
Related pairs	32.20±1.40	31.67±1.40	0.27	0.79	0.06	36.91±1.48	36.59±1.48	0.26	0.80	0.05
Unrelated pairs	20.87±1.40	19.20±1.40	0.84	0.40	0.18	26.35±1.48	26.09±1.48	0.21	0.83	0.04
*Retest*										
Related pairs	31.77±1.45	28.97±1.45	1.37	0.18	0.31	36.35±1.55	36.35±1.55	0.00	0.99	0.00
Unrelated pairs	17.43±1.45	13.00±1.45	2.17	0.03	0.49	25.59±1.55	23.82±1.55	1.39	0.17	0.28
*Change*										
Related pairs	−0.43±0.52	−**2.70±0.52** b	3.10	0.002	0.58	−0.56±0.38	−0.24±0.38^b^	0.63	0.53	0.15
Unrelated pairs	−**3.43±0.52** a	−**6.20±0.52** b	3.78	0.001	0.71	−**0.76±0.38** a	−**2.26±0.38** b	2.90	0.01	0.68

Means and standard errors were estimated from mixed model analyses.

*d* = Cohen’s *d.*

Changes that were statistically different from 0 are in bold.

a Significant contrasts in changes across sleep and across nap.

b Significant contrasts in changes across 12 hours (experiment 1) and 2 hours (experiment 2) of wakefulness.

**Figure 1:**
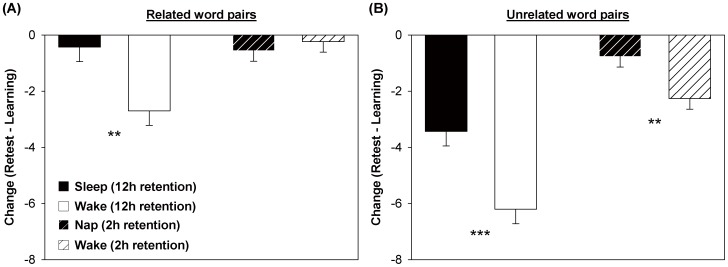
Change in paired-associate performance across the retention interval. *(* ***A*** *)* Forgetting of semantically related word pairs over a 12-hour retention interval was reduced by nocturnal sleep (black bar) relative to wakefulness during the day (white bar). In contrast, a daytime nap (black hatched bar) had no such effect on forgetting over a 2-hour retention interval compared to wakefulness (white hatched bar). *(* ***B*** *)* For semantically unrelated word pairs, both nocturnal sleep and daytime napping attenuated forgetting over the retention intervals. ** *P*<0.01; *** *P*<0.001.

Across the retention interval, forgetting was greater for unrelated than for related word pairs (Relatedness: *F_1,58_* = 46.32, *P*<0.001). The sleep group showed less forgetting than the wake group (Group: *F_1,58_* = 20.67, *P*<0.001). This beneficial effect of nocturnal sleep was similar for both kinds of materials (Relatedness×Group interaction: *F_1,58_* = 0.27, *P* = 0.60; Figure 1A & B).

### Experiment 2: daytime nap

Learning performance was better for related word pairs (Relatedness; *F_1,66_* = 116.94, *P*<0.001), but was similar in the nap and the wake conditions (Condition: *F_1,32_* = 0.13, *P* = 0.72; Relatedness×Condition interaction: *F_1,66_* = 0.00, *P* = 0.98; Table 1). Prior exposure to the task did not influence learning (Period: *F_1,32_* = 1.97, *P* = 0.17).

Forgetting across the retention interval was more prominent for unrelated word pairs (Relatedness: *F_1,66_* = 11.67, *P* = 0.001; Table 1). A post-learning nap alleviated the forgetting of unrelated word pairs (Figure 1B), but had no effect on related ones (Condition×Relatedness interaction: *F_1,66_* = 7.76, *P* = 0.01; Condition: *F_1,32_* = 2.17, *P* = 0.15; Figure 1A). Forgetting was not affected by prior exposure to the task (Period: *F_1,66_* = 0.01, *P* = 0.95).

Post-learning nap lasted for 64.1 minutes on average and consisted mainly of Stage 2 sleep. Neither nap duration nor macrostructure was associated with performance change over the retention interval (*P*>0.38; Table 2).

**Table 2:** Means and standard deviations of nap macrostructure and Pearson correlations with memory consolidation.

	Mean ± SD	Related word pairs		Unrelated word pairs	
	(min)	*r_32_*	*P*	*r_32_*	*P*
TST	64.1±14.0	−0.09	0.62	0.02	0.92
Stage 1	4.7±5.0	0.10	0.56	0.09	0.60
Stage 2	27.2±14.1	0.09	0.63	0.16	0.38
SWS	20.2±14.0	−0.12	0.48	−0.15	0.40
REM	12.0±12.5	−0.10	0.58	−0.02	0.89

*r* = Pearson *r.*

TST = total sleep time.

SWS = slow wave sleep.

REM = rapid eye movement sleep.

### Comparing the effects of nocturnal sleep and daytime napping

For related word pairs, the change in the number of correct recalls was significantly less than 0, and thus, forgetting was statistically significant, across 12 hours but not 2 hours of wakefulness (Table 1). More forgetting across longer wake periods (*t_62_* = 3.92, *P*<0.001) indicated that even for these pre-existing semantic associations, memory became vulnerable with increasing duration of wakefulness. In contrast, forgetting of related word pairs across a 12-hour interval involving nocturnal sleep and across a 2-hour period involving a daytime nap was similar (*t_62_* = 0.20, *P* = 0.84; Table 1). Hence, post-learning nocturnal sleep benefited related word pairs more than daytime napping (*d* = 0.58 and 0.15; Table 1).

In contrast, for the novel semantic associations of unrelated word pairs, nocturnal sleep and daytime napping both had medium effects on their consolidation based on conventional metrics (*d* = 0.71 and 0.68; Table 1). Forgetting of these more unusual pairings was greater over 12 than over 2 hours, and this was true for both wake (*t_62_* = 6.25, *P*<0.001) and nocturnal sleep/daytime nap retention (*t_62_* = 4.24, *P*<0.001; Fig. 1B).

Overall, post-learning sleep at night and napping during the daytime had greater facilitative effects on the memory for unrelated than for related word pairs (Table 1).

## Discussion

Nocturnal sleep was found to have greater facilitative effects on memory consolidation for related word pairs than daytime napping, but they benefited unrelated word pairs equally. These results suggest that both nocturnal sleep and daytime napping preferentially facilitates the offline processing of materials that are prone to forgetting. The pre-existing semantic associations of related word pairs are resilient to forgetting over a short wake period; thus, physiological state after learning did not influence performance change. However, when wakefulness was extended, these pre-existing associations became vulnerable. The novel associations of semantically unrelated word pairs were fragile even over short intervals. For these novel associations, nocturnal sleep and daytime napping after learning facilitated the consolidation process, thereby reducing forgetting. We cannot differentiate an active from a passive role of sleep in offline memory processing [14]. However, mounting evidence shows that sleep does not merely passively protect memory from interference [11]; instead, during sleep, recently encoded memory is reactivated [15], [16], [17] and is transferred from its temporary hippocampal store to the neocortex for more permanent storage [18], [19].

Adding to a recent report of greater improvement in a motor sequence learning task across nocturnal sleep than across a daytime nap which did not immediately follow learning [20], we showed for the first time that compared to daytime napping immediately after learning, nocturnal sleep had greater beneficial effects on the offline processing of recently encoded declarative memory, particularly those for semantically related word pairs.

Furthermore, extending an earlier observation of greater benefits of post-learning nocturnal sleep on unrelated than on related word pairs [3], we showed that daytime napping also had the same differential effects. These findings are consistent with our proposal for greater nocturnal sleep and daytime napping effects on the consolidation of vulnerable materials, as well as earlier findings for the importance of sleep in consolidating weak semantic associations [21]. Although a recent study of retrieval-induced forgetting has shown that overnight SWS is correlated with memory for materials more likely to be recalled, while REM reduces forgetting of materials more vulnerable to forgetting [22], we did not find such associations for a post-learning daytime nap.

The prefrontal cortex is more engaged in the encoding of semantically unrelated than related word pairs [23]. Hence, tagging [24] of these novel associations via the prefrontal-hippocampal systems at learning may contribute to the stronger benefits of nocturnal sleep and daytime napping on semantically unrelated materials.

Whether the greater benefits of nocturnal sleep than daytime napping are due to differences in sleep duration and structure or time of day remains to be examined. However, the non-significant associations of post-nap performance change with nap duration and macrostructure reported here and previously [4], [25] rule out the first possibility.

This study had two limitations. Learning performance was better in the nap condition in experiment 2 than the sleep group in experiment 1 (related word pairs: *t_62_* = 2.29, *P* = 0.03; unrelated word pairs: *t_62_* = 2.67, *P* = 0.01). It was also better in the wake condition in experiment 2 than the wake group in experiment 1 (related word pairs: *t_62_* = 2.40, *P* = 0.02; unrelated word pairs: *t_62_* = 3.36, *P* = 0.001). This was likely due to superior learning in the afternoon relative to the morning and the evening. Nevertheless, differences in learning performance were controlled for in the indices of memory consolidation which were derived by subtracting performance in the learning session from that in the retest session.

Despite the differences in study design and stimuli between our two experiments, they were, in fact, similar in many ways, such as demographic characteristics of the participants, criteria for participant selection (except for the frequent napper requirement in experiment 2), enforcement of habitual sleep schedules the night prior to the experiment, numbers of semantically related and unrelated word pairs, and task instructions. The experimental designs adopted in some previous studies [20], [22] afford a direct statistical comparison between the nocturnal sleep and the daytime napping effects. Nevertheless, the differences across existing studies in memory tasks, stimuli, and study design (e.g. delays between learning and retrieval, and their timing in relation to sleep) are considerable, increasing the difficulty in generalizing findings beyond single studies. Future studies may adopt a meta-analytic approach, using existing findings to quantify the effect sizes of nocturnal sleep and daytime napping, and to determine whether these effects are moderated by memory tasks, the nature of the stimuli, and study design.

## References

[1] Rasch B, Born J (2013) About sleep’s role in memory. Physiol Rev 93: 681–766. 23589831 10.1152/physrev.00032.2012 PMC3768102

[2] Jenkins JG, Dallenbach KM (1924) Oblivescence during sleep and waking. Am J Psychol 35: 605–612.

[3] Payne JD, Tucker MA, Ellenbogen JM, Wamsley EJ, Walker MP, et al (2012) Memory for semantically related and unrelated declarative information: the benefit of sleep, the cost of wake. PLoS One 7: e33079. 22457736 10.1371/journal.pone.0033079 PMC3310860

[4] Tucker MA, Hirota Y, Wamsley EJ, Lau H, Chaklader A, et al (2006) A daytime nap containing solely non-REM sleep enhances declarative but not procedural memory. Neurobiol Learn Mem 86: 241–247. 16647282 10.1016/j.nlm.2006.03.005

[5] Tucker MA, Fishbein W (2008) Enhancement of declarative memory performance following a daytime nap is contingent on strength of initial task acquisition. Sleep 31: 197–203. 18274266 10.1093/sleep/31.2.197 PMC2225575

[6] Gais S, Born J (2004) Declarative memory consolidation: mechanisms acting during human sleep. Learn Mem 11: 679–685. 15576885 10.1101/lm.80504 PMC534696

[7] Plihal W, Born J (1997) Effects of early and late nocturnal sleep on declarative and procedural memory. J Cogn Neurosci 9: 534–547. 23968216 10.1162/jocn.1997.9.4.534

[8] Barrett TR, Ekstrand BR (1972) Effect of sleep on memory: III. Controlling for time-of-day effects. J Exp Psychol 96: 321–327. 4345763 10.1037/h0033625

[9] Furstenberg CT, Sebrechts MM, Seamonm JG (1987) Accessing associative strength in cued recall and pair recognition. Am J Psychol 100: 239–251.

[10] Nelson DL, McEvoy CL, Schreiber TA (1998) The University of South Florida word association, rhyme, and word fragment norms. http://www.usf.edu/FreeAssociation/. 10.3758/bf03195588 15641430

[11] Ellenbogen JM, Hulbert JC, Stickgold R, Dinges DF, Thompson-Schill SL (2006) Interfering with theories of sleep and memory: sleep, declarative memory, and associative interference. Curr Biol 16: 1290–1294. 16824917 10.1016/j.cub.2006.05.024

[12] Rechtschaffen A, Kales A (1968) A Manual of Standardized Terminology, Techniques and Scoring System for Sleep Stages of Human Subjects. Los Angeles: BIS/BRI, UCLA. 10.1046/j.1440-1819.2001.00810.x 11422885

[13] Cohen J (1988) Statistical power analysis for the behavioral sciences. Hillsdale, NJ: Lawrence Erlbaum Associates.

[14] Ellenbogen JM, Payne JD, Stickgold R (2006) The role of sleep in declarative memory consolidation: Passive, permissive, active or none? Curr Opin Neurobiol 16: 716–722. 17085038 10.1016/j.conb.2006.10.006

[15] Peigneux P, Laureys S, Fuchs S, Collette F, Perrin F, et al (2004) Are spatial memories strengthened in the human hippocampus during slow wave sleep? Neuron 44: 535–545. 15504332 10.1016/j.neuron.2004.10.007

[16] Rasch B, Buchel C, Gais S, Born J (2007) Odor cues during slow-wave sleep prompt declarative memory consolidation. Science 315: 1426–1429. 17347444 10.1126/science.1138581

[17] Wilson MA, McNaughton BL (1994) Reactivation of hippocampal ensemble memories during sleep. Science 265: 676–679. 8036517 10.1126/science.8036517

[18] Gais S, Albouy G, Boly M, Dang-Vu TT, Darsaud A, et al (2007) Sleep transforms the cerebral trace of declarative memories. Proc Natl Acad Sci U S A 104: 18778–18783. 18000060 10.1073/pnas.0705454104 PMC2141853

[19] Takashima A, Petersson KM, Rutters F, Tendolkar I, Jensen O, et al (2006) Declarative memory consolidation in humans: a prospective functional magnetic resonance imaging study. Proc Natl Acad Sci U S A 103: 756–761. 16407110 10.1073/pnas.0507774103 PMC1334654

[20] Doyon J, Korman M, Morin A, Dostie V, Hadj Tahar A, et al (2009) Contribution of night and day sleep vs. simple passage of time to the consolidation of motor sequence and visuomotor adaptation learning. Exp Brain Res 195: 15–26. 19277618 10.1007/s00221-009-1748-y PMC2752878

[21] Drosopoulos S, Schulze C, Fischer S, Born J (2007) Sleep’s function in the spontaneous recovery and consolidation of memories. J Exp Psychol Gen 136: 169–183. 17500644 10.1037/0096-3445.136.2.169

[22] Baran B, Wilson J, Spencer RM (2010) REM-dependent repair of competitive memory suppression. Exp Brain Res 203: 471–477. 20401652 10.1007/s00221-010-2242-2 PMC3259851

[23] Sandrini M, Cappa SF, Rossi S, Rossini PM, Miniussi C (2003) The role of prefrontal cortex in verbal episodic memory: rTMS evidence. J Cogn Neurosci 15: 855–861. 14511538 10.1162/089892903322370771

[24] Stickgold R, Walker MP (2013) Sleep-dependent memory triage: Evolving generalization through selective processing. Nat Neurosci 16: 139–145. 23354387 10.1038/nn.3303 PMC5826623

[25] Lahl O, Wispel C, Willigens B, Pietrowsky R (2008) An ultra short episode of sleep is sufficient to promote declarative memory performance. J Sleep Res 17: 3–10. 18275549 10.1111/j.1365-2869.2008.00622.x

